# Creating boundaries to empower digital health technology

**DOI:** 10.1192/bjo.2018.37

**Published:** 2018-07-09

**Authors:** Honor Hsin, John Torous

**Affiliations:** Clinical Psychiatrist, Verily Life Sciences, South San Francisco, California, USA; Clinical Psychiatrist, Departments of Psychiatry and Clinical Informatics, Beth Israel Deaconess Medical Center, Harvard Medical School, Boston, Massachusetts, USA.

**Keywords:** Smartphones, mHealth, digital, psychiatry

## Abstract

The potential of digital health tools such as smartphones and sensors to increase access to and enhance delivery of healthcare is well known. However, a lack of regulation and delineation between those technologies seeking to offer direct clinical diagnostics and treatments and those involving clinical care enhancements or direct-to-consumer resources has led to patient and clinician confusion about the appropriate use and role of digital health. Here, we propose that creating boundaries and better defining the scope of digital health technology will advance the field through matching the right use cases with the right tools. We further propose that ethical clinicians, as stewards of standard of care, are well suited to uphold these boundaries and to safeguard best practices in digital health.

**Declaration of interest:**

H.H. is an employee of Verily Life Sciences and owns equity in this company. The views expressed here are those of the authors and are not official views of Verily Life Sciences.

With over 250 000 mobile health applications available today, at least 10 000 of which target mental health conditions,^1^ digital health technology has become an emergent force in modern clinical care. According to industry reports, mental health is the most common focus of disease-specific mobile applications, constituting 29% of all chronic condition management apps in 2015.^2^ This digital health movement embodies the hope that decades of progress in technical capabilities, data analytics and knowledge of the consumer experience can improve clinical outcomes, enhance well-being and democratise healthcare. However, to realise this potential it is now critical, even if paradoxical, to draw new boundaries in the seemingly boundless world of digital health.

The current lack of boundaries in digital health is a logical result of product development driven by patient and entrepreneurial perspectives. Patients experience health along a continuum from wellness to disease, and the digital opportunity to blend care and move interventions from reactive to proactive carries potential benefits for improved outcomes, healthcare costs and user satisfaction. In contrast to this continuous patient experience, however, clinical practice remains dichotomous with respect to professional responsibility, medical necessity, legal liability and reimbursement. Tensions arise, therefore, when digital health attempts to bridge the wellness-to-disease divide too aggressively, or when boundaries between clinical responsibility and consumer self-care become blurred. In 2016, the software company Lumos Labs, for example, was fined US$2 million by the Federal Trade Commission for falsely claiming that its ‘brain training’ games prevent clinical deterioration in dementia, brain injury and mental health disorders.^3^ A review of mobile applications for suicide prevention found a product that advocated self-harm and drug use as coping skills for suicidal ideation.^4^ These examples and others raise spectres of mistrust, lack of transparency and potential for patient harm – all possible barriers toward wider adoption of digital technology in healthcare.^1^^,^^5^

There is an urgent need to unite the potential of digital health with the fundamental ethics of clinical practice, and to encourage innovation while protecting both the future of digital health and the trust it requires to engage patients and clinicians. By acknowledging the limitations of digital health and establishing appropriate boundaries of use, the field can realise the true potential of what technology offers, while concurrently curtailing unfounded claims. Toward this end, we propose a practical taxonomy accessible to clinicians comprising three categories of digital health uses to illustrate these boundaries: (a) treatment and diagnosis, (b) care enhancement, and (c) resources. We propose that adopting this rubric at the level of clinical judgement in direct patient care, as opposed to abstract academic and evolving regulatory standards, can enable proper empowerment of digital health technology by harnessing the utility of decision-guiding frameworks that clinicians already use in practice today – namely, the appropriate balancing of clinical harms and risks, and degree of integration with the therapeutic relationship.

## Treatment and diagnosis

Digital technologies can become direct tools of clinical practice. Examples include monitoring or therapeutic devices that encapsulate clinical data used for direct medical decision-making, such as a wearable glucose sensor or digital insulin pump, respectively. Quality of evidence for these clinical uses must be held to publication-level benchmarks of efficacy or validation to gold-standard outcomes, owing to the high potential for clinical harm in the event of malperformance. Recent meta-analysis of apps directed at management of depressive symptoms,^6^ anxiety disorders^7^ and self-harm^8^ all report similar results, with a small evidence base derived from mostly heterogeneous pilot studies. As in other areas of healthcare that are working to create standards for smartphone apps,^9^ the mental health community will need to unite to determine what level and standard of evidence is sufficient for clinical use.

However, while the evidence base remains nascent and standards have not yet been established, maintaining a clear boundary between those digital technologies that process data of vital treatment purpose versus those that make unsupported claims of such is crucial for allowing both patients and clinicians to find and utilise only validated interventions for such critical medical roles. In mental health, examples of diagnostics with potential for patient harm could be a cognitive function assessment that claims the ability to diagnose dementia, or a voice assessment tool that claims the ability to diagnose schizophrenia – in these cases, the direct link to subsequent clinical decision-making of grave consequence necessitates a high degree of published evidence prior to clinical implementation. Just as new devices such as a seizure detection watch-like device went through formal clinical studies prior to receiving clinical approval from the European Medicines Agency and United States Food and Drug Administration (FDA),^10^ new software in the form of apps that makes clinical claims must be well validated.

The danger of unsupported claims is reflected in the first version of the UK's National Health Service App Library that was abruptly discontinued when apps on the site were found to not meet basic security standards or possess any clinical evidence. Research has demonstrated that star ratings or number of downloads on app marketplaces do not correlate with the clinical quality or utility of apps.^11^ In the USA, the FDA has announced a novel precertification programme, where app makers are pre-approved to release FDA-cleared health apps based on their track record of patient safety, product quality, clinical responsibility, cybersecurity responsibility and proactive culture.^12^ It is possible that this and other potential approaches to regulatory governance may help guide standards for future clinical claims.

## Care

Just because an app may not diagnose or treat an illness does not mean it is less valuable. There must be recognition of the equally important need for technology to improve the efficiency or experience of clinical care. Rather than constituting the clinical treatment process, technology here can enhance it for patients, providers or health systems. Support tools range from personal symptom monitoring and user engagement products to care coordination and collaboration software. Wellness mobile applications, serving functions such as stress management or weight loss, also fall into this category if used by clinicians to integrate with and thereby augment standards of care.

Quality of evidence for care technologies has traditionally been less stringent and more variable here than for the treatment/diagnosis category. Depending on degree of digital autonomy, supporting evidence may not be needed. Other sources of evidence may range from naturalistic or feasibility studies to randomised controlled trials, and a variety of outcomes may be examined, from clinical and functional endpoints to cost-effectiveness and care efficiency.^13^ Regulatory oversight in this broad category of care uses is less clear; therefore, careful provider oversight and patient data stewardship are crucial to safe implementation in practice.^14^ Evaluating the safety and utility of apps in this care category requires careful clinical judgement and shared decision-making with the unique patient and use case at hand. For example, a simple symptom-monitoring app can be used in numerous ways, including tracking response to a medication, predicting risk of relapse or increasing awareness of symptoms. Deferring critical thinking about apps to third-party rating systems that offer point-based scoring or ‘expert’ reviews has been shown to be unreliable^15^ and of questionable validity.^16^

## Resources

Digital technologies can also be resources that improve access to medical care or improve health outside of the therapeutic relationship. Examples include wellness, education, peer counselling and referral services, which may play an important part in enhancing patient self-agency. On the other hand, supporting evidence for this effect and regulatory oversight of products are, by circumstance, the most minimal and least standardised of the three categories. Disclaimers on intended use of resources are sometimes the only method of protection against harmful user outcomes. While there may be less need for oversight given the less stringent standards for health claims, digital health tools in this category can nonetheless be very effective. A thoughtful example of this category is the conversational app Woebot, designed with cognitive–behavioural therapeutic principles in mind to assist users in recognising and challenging cognitive distortions. Woebot seeks to disseminate useful skills, while clearly defining that these are not treatment claims and not a substitute for professional care.^17^

Recognising the intended role and proper categorisation of these resources is critical for allowing this ecosystem to flourish within the proper scope without inappropriate regulation. Instead, many mental health apps currently available for consumer download market treatment or care claims, but often include in small print in the terms and conditions that they should legally be considered only a resource.^18^ This dichotomy between marketing and legal claims is confusing to patients and clinicians and obfuscates the potential of all apps.

## Empowering digital health

Clinicians, by virtue of professional commitments to evidence-based practice, are well positioned to identify these three boundaries and also their violations (Table 1). With this rubric, the mistake made by Lumos Labs in making treatment claims for a resource product (consumer wellness) becomes readily apparent.^3^ There may also be examples where digital treatments are more appropriately grouped as resources and are therefore too constrained under the treatment/diagnosis categories, such as a mobile app for mindfulness. In these cases, recommending vetted products as digital resources outside clinical care may offer greater population benefits, better realise the intended use of these products, and avoid ethical and legal boundary crossings of treatment- and care-level gains.

**Table 1:** A practical taxonomy for digital health

Category:	Treatment and diagnosis	Care enhancement	Resources
Potential for clinical harm if misused or malfunctioned?	High	Medium	Low
Clinical evidence required prior to use?	Gold-standard efficacy/validation	Variable	None
Protected by therapeutic relationship?	Yes	Yes	No

However, in areas such as mental health and chronic disease management, the boundaries between wellness, care and treatment may be blurred.^5^ Psychotherapy mobile applications such as those offering insomnia cognitive–behavioural therapy pose particular challenges, as many efficacy trials do not appropriately control for the ‘digital placebo’ effect,^19^ and therefore treatment claims may be difficult to make. More recent research efforts have begun to address this issue by offering participants in control groups of studies ‘inactive’ apps such as symptom trackers. A recent study featuring a placebo app version of the popular mindfulness app called Headspace found that the placebo version resulted in similar gains in mindfulness as the actual app – suggesting that the evidence necessary to regulate these apps remains evolving and complex.^20^

Without changes in regulatory guidance, however, most psychotherapy applications would fall under the category of care (if integrated under the supervision of a clinician) or resources (if provided outside it). Some may feel that even as resources, there may still be a need for some information regarding efficacy and safety claims, much as the current dietary supplement industry offers some facts on labels. In addition, of course, some may use an app intended to be a resource as a care tool, just as today many interventions and uses of medications in care are ‘off-label’. Yet, the goal of the proposed framework is not to categorise every digital health innovation perfectly, but rather to guide rational and informed decision-making around the appropriate role and concomitant level of supporting evidence and clinical protections necessary to back such claims.

Currently, without these boundaries, there is little to guide a clinician and it is possible that a resource app may be recommended for care or treatment. The nascence of these apps means that there is no case law to understand what legal liability there may be for adverse outcomes related to apps. It also means that there is little research evidence to guide informed decision-making or categorisation for most apps. There is a need for not only more research on mental health apps but also more standardisation of research with common clinical, usability and validated psychometric measures assessed across different apps, populations and use cases. Clear standards for data collection, monitoring and governance are also essential for fostering trust and transparency in digital health.^1^ New standards and types of research methodology for understanding the value of studies that demonstrate personal health benefits with apps beyond randomised controlled studies are also necessary.^21^ New partnerships with patients to create digital tools that support both sides of the therapeutic relationship are also sorely needed.^22^

Beyond apps, the same proposed framework is applicable to other innovative technologies such as virtual reality or therapeutic video games. As the age of digital health technology dawns in modern medicine, evidence-based practitioners are uniquely situated to assume a leadership role in drawing boundaries between technology uses, factoring in clinical risk considerations in the context of standard of care. Only with this leadership and the establishment of rational boundaries can the potential of digital technology move towards the boundless applications often discussed but rarely realised today. A first step towards such leadership involves education and learning more about the risk, benefits, uses and current knowledge. We point readers to online resources such as the Australian Black Dog Institute's website (https://www.blackdoginstitute.org.au/), the American Psychiatric Association website on apps (https://www.psychiatry.org/psychiatrists/practice/mental-health-apps/app-evaluation-model), and the UK's app library website (https://apps.beta.nhs.uk/) as examples of places to begin.
